# Gastroparesis secondary to a demyelinating disease: a case series

**DOI:** 10.1186/1471-230X-7-3

**Published:** 2007-01-31

**Authors:** Savio C Reddymasu, John Bonino, Richard W McCallum

**Affiliations:** 1 Department of Medicine, Kansas University Medical Center, Kansas City, KS, USA

## Abstract

**Background:**

Gastroparesis has a number of etiologies. The main ones are secondary to a complication from diabetes mellitus, related to post vagotomy or post gastric surgical resections, or idiopathic when the etiology is unclear. Gastroparesis secondary to a demyelinating disease of the brain is unusual.

**Case presentation:**

A 22-year-old woman was referred for acute onset of intractable nausea and vomiting. She also had cerebellar deficits, dysphagia and paresthesias. Magnetic resonance imaging (MRI) of the brain revealed an isolated area of demyelination in the medullary region. Another 24-year-old woman had a similar presentation with right hemiplegia and MRI of the brain revealed a distal medullary region. Both these patients had an abnormal gastric emptying test. Gastroparesis and neurological deficits improved with intravenous corticosteroids. While the former patient has had no further recurrences, the latter patient developed multiple sclerosis within three months of presentation.

**Conclusion:**

A demyelinating disease is a rare cause gastropareis, but should be suspected when symptoms of gastroparesis are associated with neurological deficits. MRI might help in the diagnosis and intravenous coriticosteroids can address the underlying disease process and improve gastric emptying, especially when used early during the course of the disease.

## Background

Gastroparesis is a condition characterized by evidence of gastric retention in the absence of any mechanical obstruction. Symptoms usually include nausea, vomiting, post prandial fullness, and abdominal pain [1]. Diabetes Mellitus is the commonest cause of gastroparesis, other etiologies include idiopathic, and post surgical usually secondary to a vagotomy [2]. A primary neurological basis for gastroparesis is unusual. We present a series of two patients with gastroparesis due to a demyelinating disease.

## Case presentation

### Case 1

**Figure 1:**
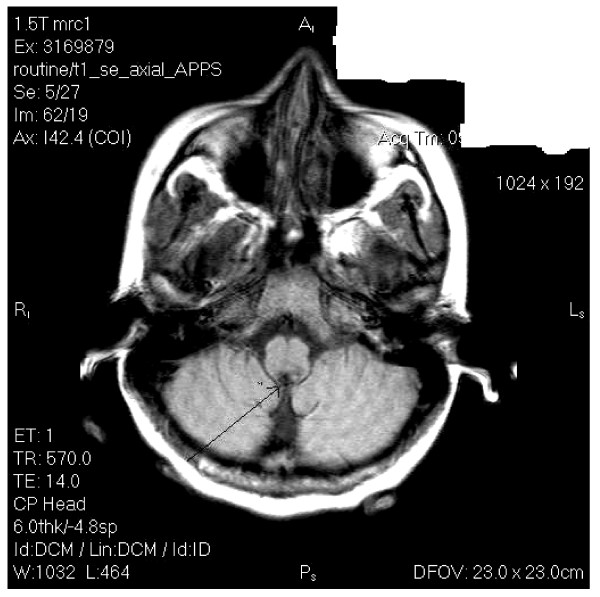
Demyelinating lesion of the brainstem at presentation.

**Figure 2:**
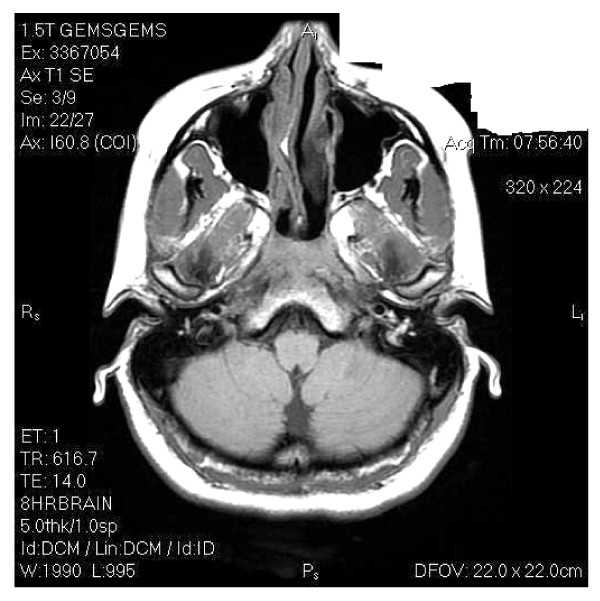
Resolution of the lesion after treatment with corticosteroids.

A 22-year old woman was seen in the emergency department with acute onset of intractable nausea and vomiting. Nausea and vomiting were predominantly post-prandial and seemed to respond partially to metoclopramide. Over the next few weeks she also developed dysphagia, blurry vision, paresthesias involving her lower and upper extremities, and problems with balance. Physical exam was significant for nystagmus on lateral gaze, quadriparesis, absent deep tendon reflexes in all four extremities, a negative plantar reflex, and a positive finger-nose test. No history of vertigo was obtained, cranial nerves were intact, and fundoscopic examination was normal. Her hospital course was complicated by renal failure secondary to dehydration, which subsequently resolved. She had a jejunal feeding tube (J-tube) placed for nutritional support. Laboratory analysis was significant for elevated creatinine initially which later resolved. Cerebrospinal fluid (CSF) analysis was remarkable only for oligoclonal bands (OB's). Magnetic resonance imaging (MRI) of the head and the cervical spine revealed a focal area of demyelination near the cervico-medullary junction (Figure 1). There was an interval increase in the size of this lesion over the next few weeks. A scintigraphic four-hour gastric emptying test (GET) was performed. The patient consumed a low fat (2%) Eggbeater meal (255 kcal) labeled with 1mCi99Tc sulfur colloid and 170 cc of water. 46% of the meal was retained at the end of 4 hours (Normal: < 10% retention at the end of 4 hours) Upper endoscopy (EGD) was negative for gastric outlet obstruction. As the patient's symptoms were worsening intravenous methylprednisolone at a dose of 1 gram daily for 2 weeks was instituted followed by a prednisone taper resulted in improvement in nausea, vomiting, and other neurological deficits. The total duration of corticosteroid use was for two months. A repeat MRI after treatment showed resolution of the lesion in the medulla (Figure 2). A follow up GET was normal with 8 % retention of the radionuclide meal at the end of four hours. After the patient's oral intake improved and she could keep up with her nutritional requirements, the J-tube was discontinued after 6 months of initial presentation. At the 1-year follow up visit the patient had no neurological or gastrointestinal (GI) complaints, was off all medications, actively employed, and functioning with no complaints.

### Case 2

A 24 year old woman was referred to our hospital with symptoms of persistent post-prandial nausea, vomiting, new onset right sided weakness, and paresthesias. Symptoms started 2 weeks prior to presentation. Physical exam was positive for facial nerve palsy on the right side, reduced motor strength in the right upper and lower extremities, absent deep tendon reflexes on the right side, plantar- flexor response, abnormal finger-nose test, and a broad-based unsteady gait. Fundoscopic examination was normal. EGD was negative. GET performed by the previously described technique was conclusive for gastroparesis with 83% retention of food at the end of four hours. MRI revealed demylenating lesions in distal medulla, extending caudally through the cervico-medullary junction. The patient was started on 1 gram of methylprednisolone daily intravenously for 10 days, followed by a tapering dose of oral prednisone and this resulted in a gradual improvement of her nausea, vomiting, and neurological symptoms. GET repeated after 10 days showed remarkable improvement with only 13% retention of food at the end of 4 hours. Three months later, she developed additional neurological symptoms and was diagnosed with multiple sclerosis (MS). No recurrence of GI symptoms was reported.

## Discussion

The patients presented above had an acute onset of neurological deficits and gastroparesis associated with a lesion in the medulla. Treatment with corticosteroids led to resolution of gastroparesis. Simultaneous improvement of symptoms of gastroparesis and size of the brain lesion support the primary neurological etiology for gastroparesis. The demyelinating disease could have affected the vagal nerve nuclei in these patients, which explains their GI complaints. Though, corticosteroids can help nausea and vomiting especially when associated with chemotherapy, they do not improve gastric emptying [3,4]. The role for corticosteroids in MS is to treat of acute symptomatic relapses of MS [5,6]. Treatment with corticosteroids in other demyelinating conditions such as acute optic neuritis has also been shown to reduce the onset of MS [7]. The same might apply in this particular scenario also. Prokinetic medications, such as metoclopramide and domperidone do not affect the course of the disease in MS, unless patients have symptoms of nausea and vomiting.

An intact vagal nerve is essential for gastric emptying. The vagus nerve affects gastric motility by coordinating the appearance of the gastric migrating motor complex (MMC) in the fasting state and help in the generation of the digestive motor pattern after eating a meal, which leads to gastric emptying. Vagal blockade has been associated with impairment of MMC and delayed gastric emptying of a solid meal resulting in symptoms of nausea, vomiting, postprandial fullness, abdominal pain, and weight loss [8]. Another contributing factor for the delayed gastric emptying observed in our patients might be the unopposed adrenergic drive secondary to demyelination of the vagal nerve nuclei [9]. A further possible explanation for the gastroparesis could be a disturbance to the vestibular nuclei in the brain stem region, which may lead to a delay in gastric emptying [10].

Slow gastric emptying is an infrequent complication of MS occurring many years after disease onset [11-14]. This is in contrast to the patients presented above, where they were healthy before manifesting symptoms of gastroparesis and developing a demyelinating lesion in the brainstem. A similar case report was found in literature where the disease course was very similar to the patients described above [15]. One patient (Case 1) had OB's in her CSF has been free of any recurrences to date. She fit the profile for clinically isolated demyelinating disease of the brain stem. However OB's in the CSF predict the development of MS in the future [16].

## Conclusion

Gastroparesis with neurological symptoms might indicate a demyelinating disease of the central nervous system and predict development of MS in the future. MRI of the brain is recommended in patients with neurological deficits and symptoms of delayed gastric emptying. Administration of corticosteroids early in the course of the disease seems to resolve the demyelinating process, thus improving gastroparetic symptoms and normalizing gastric emptying test results.

## Competing interests

The author(s) declare that they have no competing interests.

Financial competing interests

• In the past five years have you received reimbursements, fees, funding, or salary from an organization that may in any way gain or lose financially from the publication of this manuscript, either now or in the future? Is such an organization financing this manuscript (including the article-processing charge)? If so, please specify – **NO**

• Do you hold any stocks or shares in an organization that may in any way gain or lose financially from the publication of this manuscript, either now or in the future? If so, please specify. **NO**

• Do you hold or are you currently applying for any patents relating to the content of the manuscript? Have you received reimbursements, fees, funding, or salary from an organization that holds or has applied for patents relating to the content of the manuscript? If so, please specify. **NO**

• Do you have any other financial competing interests? If so, please specify. **NO**

## Authors' contributions

1. SCR conceptualized and drafted the manuscript

2. JB revised the manuscript for important intellectual content

3. RM is the senior author, edited the manuscript for important intellectual content and has read and approved the final version of the manuscript.

## Pre-publication history

The pre-publication history for this paper can be accessed here:
